# Supplementary material for: Proteomic Analysis Revealed the Antagonistic Effect of Decapitation and Strigolactones on the Tillering Control in Rice
Source: Plants (Basel). 2023 Dec 27;13(1):91. doi: 10.3390/plants13010091 (PMC10780617; doi:10.3390/plants13010091)
Supplement: Supplementary file 1 [file plants-13-00091-s001.zip › plants-2748512-supplementary.pdf]

Table S1 DAPs enriched in both RP-Co and GR-RP compare groups in rice bud growth regulation.

Gene Name	ID	RP-Co Fold Change	GR-RP Fold Change	Protein Name
LOC_Os04g58280	Q0J8Z3	0.358833333	0.3309	stem-specific protein TSJT1
LOC_Os06g05350	Q5VP52	2.022383333	0.354222222	WHIRLY family protein
LOC_Os03g50110	Q53RK1	2.760783333	0.376811111	transcription regulator
LOC_Os01g42080	Q5ZD97	2.734283333	0.378511111	zinc ion binding protein
LOC_Os06g04020	Q9SNN5	0.1779	0.439911111	histone H1
LOC_Os01g13060	Q8LR51	2.6675	0.450444444	Casein kinase I-like
LOC_Os03g27310	Q0JCT1	6.6345	0.468855556	Histone H3.3
LOC_Os02g05330	Q6Z2Z4	2.159933333	0.472944444	Eukaryotic initiation factor 4A-3
LOC_Os08g39820	Q6YVT3	2.952266667	0.4737	pre-rRNA-processing protein TSR2
LOC_Os05g07640	Q60DU1	2.7872	0.4804	expressed protein
LOC_Os04g58280	Q7XKE6	0.399766667	0.487944444	stem-specific protein TSJT1
LOC_Os01g73550	Q5JJV3	2.2303	0.490788889	mitochondrial-processing peptidase
LOC_Os06g47350	Q653F3	2.580916667	0.4966	RNA polymerase I specific transcription initiation factor
LOC_Os05g06140	Q65XP3	51.05308333	2.200144444	lipase, putative
LOC_Os08g38900	Q9XGP7	2.254733333	2.243144444	Tricin synthase 1
LOC_Os12g35580	Q2QP59	0.491833333	2.247488889	Riboflavin synthase, alpha subunit family protein
LOC_Os03g56310	Q7XZV8	5.27915	2.542077778	HAD superfamily hydrolase, 5'-Nucleotidase containing protein
LOC_Os01g08270	Q94EG1	2.097866667	2.798333333	amino transferase
LOC_Os11g38260	Q2R1S1	0.473483333	2.9047	Harpin binding protein 1
LOC_Os12g36210	Q2QNZ6	0.478466667	3.257744444	inhibitor I family protein
LOC_Os06g37150	Q5Z5T3	0.497316667	3.454833333	L-ascorbate oxidase
Os01g0667300	C7IWX5	9.000416667	3.481488889	
LOC_Os05g33550	Q6AVA9	0.410583333	3.760322222	methyl-binding domain protein MBD
LOC_Os03g58260	Q7Y1H9	0.387933333	3.996555556	tryptophan synthase
OJ1200_C08.101	Q7XIR3	0.393883333	4.701955556	Nucleoporin domain 1
LOC_Os01g72490	Q941Y5	0.442266667	5.551211111	LRP1, putative, expressed
OJ1006F06.6	Q8H8D9	0.1633	6.246388889	Glutathione S-transferase
LOC_Os02g56900	Q6K3E0	0.29705	6.301911111	thioredoxin family protein
